# Implementation of Fingerprint Technology for Unique Patient Matching and Identification at an HIV Care and Treatment Facility in Western Kenya: Cross-sectional Study

**DOI:** 10.2196/28958

**Published:** 2021-12-22

**Authors:** Noah Kasiiti Jaafa, Benard Mokaya, Simon Muhindi Savai, Ada Yeung, Abraham Mosigisi Siika, Martin Were

**Affiliations:** 1 Institute of Biomedical Informatics Moi University Eldoret Kenya; 2 Vanderbilt Institute for Global Health Vanderbilt University Medical Center Nashville, TN United States; 3 Department of Medicine School of Medicine Moi University Eldoret Kenya; 4 Department of Biomedical Informatics and Medicine Vanderbilt University Medical Center Nashville, TN United States

**Keywords:** biometrics, patient matching, fingerprints, unique patient identification, electronic medical record systems, low- and middle-income countries (LMICs)

## Abstract

**Background:**

Unique patient identification remains a challenge in many health care settings in low- and middle-income countries (LMICs). Without national-level unique identifiers for whole populations, countries rely on demographic-based approaches that have proven suboptimal. Affordable biometrics-based approaches, implemented with consideration of contextual ethical, legal, and social implications, have the potential to address this challenge and improve patient safety and reporting accuracy. However, limited studies exist to evaluate the actual performance of biometric approaches and perceptions of these systems in LMICs.

**Objective:**

The aim of this study is to evaluate the performance and acceptability of fingerprint technology for unique patient matching and identification in the LMIC setting of Kenya.

**Methods:**

In this cross-sectional study conducted at an HIV care and treatment facility in Western Kenya, an open source fingerprint application was integrated within an implementation of the Open Medical Record System, an open source electronic medical record system (EMRS) that is nationally endorsed and deployed for HIV care in Kenya and in more than 40 other countries; hence, it has potential to translate the findings across multiple countries. Participants aged >18 years were conveniently sampled and enrolled into the study. Participants’ left thumbprints were captured and later used to retrieve and match records. The technology’s performance was evaluated using standard measures: sensitivity, false acceptance rate, false rejection rate, and failure to enroll rate. The Wald test was used to compare the accuracy of the technology with the probabilistic patient-matching technique of the EMRS. Time to retrieval and matching of records were compared using the independent samples 2-tailed *t* test. A survey was administered to evaluate patient acceptance and satisfaction with use of the technology.

**Results:**

In all, 300 participants were enrolled; their mean age was 36.3 (SD 12.2) years, and 58% (174/300) were women. The relevant values for the technology’s performance were sensitivity 89.3%, false acceptance rate 0%, false rejection rate 11%, and failure to enroll rate 2.3%. The technology’s mean record retrieval speed was 3.2 (SD 1.1) seconds versus 9.5 (SD 1.9) seconds with demographic-based record retrieval in the EMRS (*P*<.001). The survey results revealed that 96.3% (289/300) of the participants were comfortable with the technology and 90.3% (271/300) were willing to use it. Participants who had previously used fingerprint biometric systems for identification were estimated to have more than thrice increased odds of accepting the technology (odds ratio 3.57, 95% CI 1.0-11.92).

**Conclusions:**

Fingerprint technology performed very well in identifying adult patients in an LMIC setting. Patients reported a high level of satisfaction and acceptance. Serious considerations need to be given to the use of fingerprint technology for patient identification in LMICs, but this has to be done with strong consideration of ethical, legal, and social implications as well as security issues.

## Introduction

### Background

Unique patient matching and identification across health services is an operational challenge in many health care settings [1-4]. Commonly used identifiers such as patient names, ID numbers, national ID numbers, and dates of birth are often inadequate at guaranteeing unique patient identification, and sometimes these data may not be available [5-7]. Failure to correctly match patients largely contributes to inefficiencies in care delivery, resulting in medical errors that can affect patient safety [8]. In the United States alone, an estimated average of 400,000 deaths occur annually because of medical errors [9]. The problem is even more significant in low- and middle-income countries (LMICs), with many of these countries yet to implement accurate and comprehensive person identification procedures that ensure unambiguous identification of all citizens and residents [10].

Patient identity management has relied heavily on either deterministic or probabilistic algorithms, as well as other statistical matching procedures [11,12]. Deterministic matching algorithms use exact match or comparisons between 2 fields [13]. These algorithms compare identifiers such as national IDs for each record to determine a match. Probabilistic matching, which is by far the most widely implemented technique for record matching [14], does not necessarily depend on unique identifiers [15]. This technique assigns weights to records to calculate the probability of linkage among them; it then uses the observed frequency of agreement and disagreement patterns among the records. The total linkage weight is then compared with a threshold above which pairs are considered a match [16-18]. The challenge with deterministic algorithms is their lack of scalability and the requirement for expensive customization and business rule revisions as databases grow in size [19]. Furthermore, not all probabilistic algorithms applied to the same set of circumstances yield results with the same degree of accuracy because they are based on field-specific weights rather than value-specific weights [20]. Application of statistical matching approaches is further limited where patients may have isolated sets of their information distributed across multiple disparate systems. In addition, people may not consistently use their official names, with some preferring to use abbreviations or alternative expressions. Name misspellings and name-order transpositions are also encountered [21,22].

In our own institutional experience, the evaluation of various 4-string manipulation strategies to improve the performance of probabilistic models of patient matching based on Kenyan names revealed a suboptimal specificity and positive predictive value of <50% when matching patient names from 2 independent clinical databases [23]. This was indeed a low level of performance accuracy, especially in a health care setting. It is therefore imperative that better patient-matching approaches are determined, especially as settings start to exchange, aggregate, and centralize clinical data from multiple sources. Beyond matching performance, any approach to be considered has to be relatively affordable, feasible, and acceptable in LMIC care settings.

### Use of Fingerprinting

Biometric approaches offer a potential solution to the challenge by trying to tackle some of issues identified in the traditional patient-matching algorithms [24]. The use of fingerprinting is already quite common in many LMICs, often for national-level voter registration and immigration services, among others. Fingerprinting and other biometric technologies are not without challenges. Special consideration needs to be given to the ethical, cultural, social, and legal implications of these solutions [25]. For example, some people may have concerns about the safety, encryption, and secondary use of their biometric data [26]. Studies have cited concerns by users about the hygiene of touching these devices [27-29]. Given the potential benefits of these technologies for improving patient matching and the poor performance of existing statistical patient-matching models, rigorous evaluations need to be conducted that weigh fingerprinting benefits (improving matching) against concerns by key stakeholders.

The basic principle of biometric authentication is that everyone is unique and can be identified by their intrinsic physical or behavioral traits [30]. Biometric technologies such as fingerprinting offer a potentially promising solution given that fingerprinting scanners are ubiquitous, the technology is relatively easy to acquire and use [31], the scanners require minimal database memory requirements, and there have been several demonstrated instances of their large-scale implementation and dominance in other sectors [32,33]. A fingerprint consists of a pattern of ridges and valleys in the surface of the fingertips, and its formation is related to the earlier fetal months [34]. It is the ridges and valleys that are extracted as minutiae that are converted into a template for storage in the database. The template is always retrieved and compared with a fresh scan (new template) during matching and identifications processes [35]. In this study, we set out to (1) evaluate the performance of using fingerprint biometric technology for patient matching within an LMIC setting and (2) evaluate acceptability and patient perception of using this system for patient identification and matching.

## Methods

### Study Setting and Participants

This study was conducted at care facilities served by the Academic Model Providing Access to Healthcare (AMPATH) in Western Kenya. The program offers HIV care and treatment services, with more than 100,000 patients who have tested positive for HIV currently under care. The program operates in partnership with Kenya’s Ministry of Health and with support from the United States Agency for International Development. This study was conducted at AMPATH clinical facilities located in Eldoret, Kenya. The study population included consenting patients aged ≥18 years visiting the AMPATH clinics for care. Patients aged <18 years were excluded because of the intricacies involved in obtaining their consent and in filling surveys. AMPATH uses an adaptation of the Open Medical Record System, an open source electronic medical record system (EMRS) deployed widely in LMICs [36,37]. Patient records in the system contain demographic information that includes several patient attributes, for example, name, age or birthdate, national ID number if available, and telephone number. The Fellegi–Sunter algorithm is used within the EMRS for patient matching. The Fellegi–Sunter algorithm is a weighted probabilistic-based record linkage approach that relies on matching weights to compute a maximum likelihood estimate that determines whether a record pair is a match or nonmatch [38].

### Study Design

The mUzima Fingerprint Module version 1.0 was developed to work as an integrated module within the EMRS in a client server–model architecture. The module was developed using Java Web Start (Oracle Corporation), which uses the Java Network Launching Protocol to download and run the fingerprint application locally on the client machine. This particular technology provides an easy 1-click activation of remotely hosted applications, guaranteeing application efficiency and elimination of complicated installation or upgrade procedures [39].

### Fingerprint Technology–Matching Algorithm

The fingerprint-matching technology was based on the Bozorth3 fingerprint-matching algorithm (National Institute of Standards and Technology) [40]. Bozorth3, a minutiae-based algorithm, does both one-to-one and one-to-many matching operations. The recommended Bozorth3 threshold is the integer 40. However, during the technology testing phase, test runs at a threshold of 40 often produced false positives, and after numerous tests, a threshold of 70 proved to be a better measure to work with for this study. For every patient, 3 instances of the same left thumbprint were captured, converted into templates, and stored in the database. A patient was identified positively if any of the 3 instances returned a matching value of ≥70, the set threshold; otherwise, it returned a nonmatch.

### Fingerprint Technology User Interface

**Figure 1:**
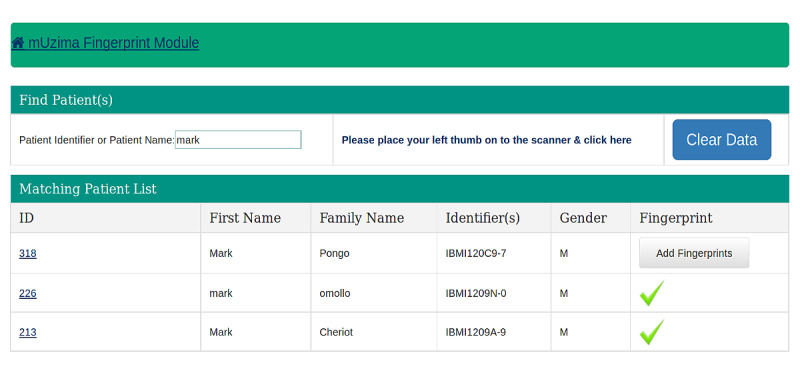
Screenshot showing the output of a patient search (“Mark”) using a probabilistic-based algorithm.

**Figure 2:**
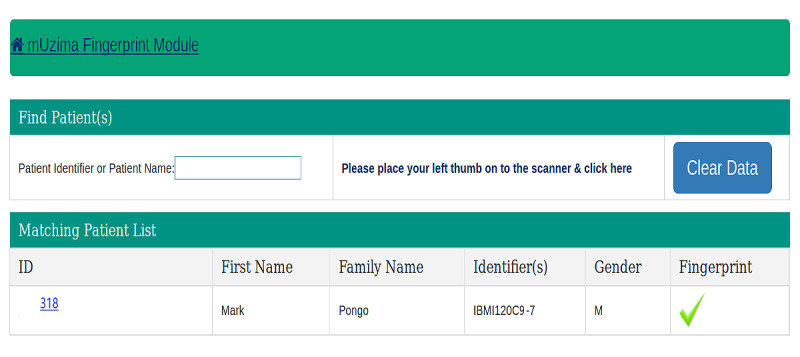
Screenshot showing the output of a patient search (“Mark”) using fingerprint technology.

The fingerprint module user interface provided 2 options for searching a patient either by their demographic information (name) or by fingerprint. A name search using the probabilistic patient-matching algorithm of the current EMRS returned all database instances with the same or similar name, with matches refined as more information was entered (Figure 1). However, when the same person was searched using their fingerprint as the identifier, a distinct and unique result was returned (Figure 2). The workflow for the fingerprint technology module is presented in Multimedia Appendix 1.

### Matching Accuracy and Time to Retrieve Records (Fingerprint Technology vs Probabilistic Matching)

The current probabilistic patient-matching method used within the AMPATH EMRS was compared with the integrated fingerprint-matching and identification module. A convenience sample of 300 patients was recruited and enrolled for the study. When the patients arrived at the clinic for the visit, they received informed consent from the study researcher (NJK), and if they consented, 3 instances of their left thumbprint were captured through the EMRS interface. The fingerprint module converted the captured prints into a template and stored them in the database for later use, to either match or retrieve records. Record retrieval was performed at different points of care within the facility, as would have been required of a provider retrieving a patient record before offering care.

The performance of the fingerprint technology was evaluated using standard measures: participants correctly identified, sensitivity, false acceptance rate (FAR), false rejection rate (FRR), and failure to enroll rate (FER). The Wald test for proportions was used to compare the accuracy of the fingerprint technology with that of the Fellegi–Sunter probabilistic patient-matching technique used within the EMRS. Time to matching and retrieval of records (measured in seconds using a timer) were compared when records were retrieved through matching of data entered by clinical personnel against those retrieved through fingerprint technology–based patient matching. A survey was also administered to evaluate participants’ acceptance and satisfaction of the fingerprint technology (Multimedia Appendix 2). The survey evaluated participants’ understanding, comfort, perception, and willingness to accept the use of fingerprint technology for patient identification.

### Fingerprint Technology Performance Measures (FAR, FRR, and FER)

Data entry and analyses were performed using STATA software (version 13.1 SE; StataCorp LLC). Age was summarized using mean and the corresponding SD. Categorical variables, for example, gender, level of education, and occupation, were summarized using frequencies and the corresponding percentages. Calculations were also performed on the standard biometric measures (Table 1).

**Table 1:** Calculated fingerprint technology performance measures.

Measure	Definition	Formula
Sensitivity	The ability of a system to correctly identify and match patients enrolled in the database	True positives/(true positives + false negatives) × 100
Specificity	The ability of the system to correctly reject patients not enrolled in the database	True negatives/(true negatives + false positives) × 100
False acceptance rate	The probability that a user who should be rejected is accepted by the system	False acceptance/total number of attempts × 100
False rejection rate	The probability that a user who should be accepted is rejected by the system	False rejection/total number of attempts × 100
Failure to enroll rate	The rate at which attempts to create a template from a scanned image are unsuccessful	False attempts/total number of attempts × 100

### Acceptability of the Fingerprint-Matching System

Acceptability of the fingerprint biometric system was derived using 3 stand-alone variables: willingness to use the fingerprint system in the future, threats to patient privacy as a result of using the biometric system, and comfort while using the system during registration. The association between categorical variables and acceptability was assessed using the Pearson chi-square test. The logistic regression model was used to assess the predictors of acceptability of the fingerprint biometric system. Odds ratios and the corresponding 95% CIs were reported. The Wald test for proportions was used to compare the level of accuracy of the fingerprint technology with the null value (level of accuracy reported of the probabilistic patient-matching approach).

This study was approved by the institutional review and ethics committee at Moi University in Eldoret, Kenya (MU/MTRH-IREC approval number FAN: IREC 1832).

## Results

### Overview

A total of 307 participants who were approached consented to the study, of whom 300 (97.7%) were registered using the fingerprint technology for enrollment into the study; the other 7 (2.3%) could not be enrolled because of difficulties in acquiring their prints, thus leading to an FER of 2.3%.

Demographic details of those enrolled are outlined in Table 2. The participants’ mean age was 36.3 (SD 12.2) years. Of the 300 participants, 174 (58%) were women and 215 (71.7%) had secondary or higher level of education (Table 2).

**Table 2:** Sociodemographic characteristics of participants (N=300).

Variable		Values
Age (years), mean (SD)		36.1 (12.3)
**Gender, n (%)**		
	Male	126 (42)
	Female	174 (58)
**Education level, n (%)**		
	Informal	10 (3.3)
	Primary	75 (25)
	Secondary	78 (26)
	Tertiary (college or university graduate)	137 (45.7)
**Occupation, n (%)**		
	Student	66 (22)
	Working or retired	125 (41.7)
	Informal employment	109 (36.3)

### Fingerprint Technology Performance

Considering the 300 appropriately scanned fingerprints, the system had a sensitivity of 89.3% (268/300) and a specificity of zero. However, the 32 unidentified fingerprints during the patient-matching exercise were included as false negatives (32/300, 10.7%; Table 3).

**Table 3:** False acceptance rate (FAR), false rejection rate (FRR), and failure to enroll rate (FER) for the fingerprint technology.

Metric	Calculation	Result (%)
FER	7/307 × 100	2.3
FAR	0/300 × 100	0
FRR	32/300 × 100	10.7
Sensitivity	268/(266 + 32) × 100	89.3
Specificity	0/(0 + 0) × 100	0

### FAR, FRR, and FER

The fingerprint technology had an FAR of 0%, signifying that the technology was able to perfectly determine that an individual was not yet enrolled into the system, returning such instances as nonmatches. However, of the 300 enrolled individuals, 32 (10.7%) were falsely considered nonmatches (FRR), signifying that some individuals who were in the system could sometimes not be matched (Table 3). The reasons for the slightly high FRR were as follows: (1) weak finger pressure on the scanner (12/32, 37%), (2) faded prints (6/32, 19%), (3) scars on the thumb (6/32, 19%), (4) scanner response failure error (5/32, 16%), and (5) low-quality images (3/32, 9%). Of these reasons, (4) and (5) were system-generated notifications, whereas the rest were manually observed and tallied by the research team during the exercise.

### Matching Accuracy and Time to Retrieve Records (Fingerprint Technology vs Probabilistic Matching)

The accuracy of the fingerprint technology compared with that of the already established accuracy levels of the EMRS that ranged from a minimum of 50% to a maximum of 70% showed a value of *P*<.001 at both minimum and maximum levels, with a power of >99%. It took an average of 3.2 (SD 1.1, range 1.0-6.8) seconds to retrieve patient records using the fingerprint technology compared with 9.5 (SD 1.9, range 6.0-13.0) seconds (*P*<.001) using the probabilistic algorithm of the AMPATH Medical Record System.

### Participants’ Perception of Biometric Systems

Of the 300 participants, only 107 (35.7%) had heard of a biometric system before; 34 (11.3%) had used a fingerprint identification system before and 11 (3.7%) a facial recognition system, whereas none had used either an eye or iris identification system or a voice-based identification system. Of the 300 participants, 255 (85%) either strongly agreed (65.7%, 197/300) or agreed (19.3%, 58/300) that a biometric system could improve identification, 30 (10%) disagreed, and 15 (5%) responded “Don’t know” to this question. Of the 300 participants, 289 (96.3%) were comfortable or very comfortable with using the fingerprint system, 290 (96.7%) expressed satisfaction with the system, and 271 (90.3%) were willing to use fingerprint technology for patient matching and identification in the future (Table 4). Most (241/300, 80.3%) of the participants did not think that technology threatened their privacy.

**Table 4:** Participants’ perception of the piloted fingerprint biometric system (N=300).

Variable			Values, n (%)
**Level of comfort during registration using fingerprint system**			
	Very comfortable	228 (76)	
	Comfortable	61 (20.3)	
	A little comfortable	5 (1.7)	
	Not comfortable	3 (1)	
	Would rather not say	3 (1)	
**General perception of the system**			
	Satisfied	290 (96.7)	
	Dissatisfied	10 (3.3)	
**Willing to use fingerprint system in future**			
	Yes	271 (90.3)	
	No	8 (2.7)	
	Don’t know	21 (7)	
**Technology threatens own privacy**			
	Yes	45 (15)	
	No	241 (80.3)	
	Don’t know	14 (4.7)	

### Acceptability of Fingerprint Matching

Acceptability of the fingerprint technology was derived from 3 variables: participants’ comfort: 96.3% (289/300), willingness to use the fingerprint technology in the future: 90.3% (271/300), and participants’ concerns regarding their privacy: 15% (45/300). The variables used to denote acceptability were further combined, that is, acceptability=yes if a participant was willing to use the fingerprint biometric system in the future, was comfortable or very comfortable using the fingerprint biometric system during registration, and did not feel that their privacy was threatened. The derived composite revealed that up to 77% (231/300) of the participants demonstrated acceptability of the fingerprint biometric system.

The Pearson chi-square test demonstrated no association between sociodemographic characteristics and acceptability of the fingerprint technology. However, the findings revealed that the respondents who agreed that the fingerprint technology offered an improved solution for patient matching were associated with the acceptability of the fingerprint technology for future use, 87.5% (202/300) versus 76.8% (53/300; *P*=.03). Similarly, the respondents who were satisfied with the fingerprint technology for identification were also associated with the acceptability of the fingerprint system for future use, 99.1% (229/300) versus 88.4% (61/300; *P*=.001; Table 5).

After adjusting for the opinion on whether the fingerprint biometric system offered an improved solution, opinion on whether the current patient identifiers (eg, IDs and patient numbers) offered an appropriate solution for identification, previous knowledge of biometric systems, and occupation, participants who had previously used fingerprint biometric systems for identification were estimated to have more than thrice the increased odds of accepting the fingerprint biometric system (odds ratio 3.57, 95% CI 1.0-11.92; Table 6).

**Table 5:** Bivariate associations of participants’ perception of biometrics and acceptability of the fingerprint technology system (N=300).

Variables		Acceptable in the future, n (%)			*P* value
		No (n=69)	Yes (n=231)		
**Have ever heard of biometric systems before**					
	No	42 (60.9)	151 (65.4)	—^a^	
	Yes	27 (39.1)	80 (34.6)	.49	
**Have ever used fingerprint biometric system before**					
	No	65 (94.2)	203 (87.9)	—	
	Yes	4 (5.8)	28 (12.1)	.14	
**Opinion on whether the current patient identifiers (eg, IDs and patient numbers) offer an appropriate solution for identification**					
	Agree or strongly agree	30 (43.5)	79 (34.2)	.16	
	Disagree or strongly disagree or don’t know	39 (56.5)	152 (65.8)	—	
**Opinion on whether biometric systems offer improved solution**					
	Agree or strongly agree	53 (76.8)	202 (87.5)	—	
	Disagree or strongly disagree or don’t know	16 (23.2)	29 (12.6)	.03^b^	
**General perception of the system**					
	Dissatisfied	8 (11.6)	2 (0.9)	—	
	Satisfied	61 (88.4)	229 (99.1)	.001^b^	

^a^Not available.

^b^Pearson chi-square test.

**Table 6:** Determinants of acceptability of the fingerprint biometric system (N=300).

Variables		UOR^a^ (95% CI)	AOR^b^ (95% CI)
**Biometric systems offer improved solution**			
	Disagree or strongly disagree or don’t know	Reference	Reference
	Agree or strongly agree	2.10 (1.06-4.16)	2.30 (1.12-4.69)
**Current patient identifiers offer an appropriate solution for identification**			
	Disagree or strongly disagree or don’t know	Reference	Reference
	Agree or strongly agree	0.68 (0.39-1.17)	0.61 (0.34-1.09)
Have ever heard of biometric systems before		0.82 (0.47-1.43)	0.51 (0.27-0.96)
Have ever used fingerprint biometric system for identification		2.24 (0.76-6.63)	3.57 (1.07-11.92)^c^
**Occupation**			
	Working or retired vs student	1.30 (0.63-2.71)	1.56 (0.73-3.33)
	Informal employment vs student	0.74 (0.36-1.51)	0.72 (0.34-1.53)

^a^UOR: unadjusted odds ratio.

^b^AOR: adjusted odds ratio.

^c^Pearson chi-square test.

## Discussion

### Principal Findings

The matching speed of the fingerprint technology was significantly lower and better than that of the demographic-based retrieval matching technology, that is, 3.2 (SD 1.1) seconds versus 9.5 (SD 1.9) seconds (*P*<.001). The system operated in a mode where no imposter was falsely considered a match in 300 attempts. However, 10.7% (32/300) of the genuine attempts were falsely considered as nonmatches (FAR 0/300 = 0.0% at an FRR 32/300 = 10.7%). The participants’ acceptability of the technology was demonstrated with most (289/300, 96.3%) of them indicating that they were comfortable with the technology and 90.3% (271/300) being willing to use the technology in the future. Although concerns about performance and acceptability of biometric technologies, especially fingerprint technology, are often discussed, few studies have comprehensively evaluated this technology in LMICs, despite fingerprint technology being widely available and affordable in these settings. Existing studies have largely described development of fingerprint technology systems without reporting on the performance using standard measures [41,42]. The few that have evaluated performance have not looked at the perceptions and acceptability of these systems [43]. To our knowledge, this is the first study to comprehensively evaluate performance, acceptability, and attitudes toward the use of fingerprint technology in an LMIC setting. The accuracy of the fingerprint technology–matching algorithm was significantly higher than that of the existing Fellegi–Sunter probabilistic algorithm. This is in line with the previous demonstration of the accuracy of fingerprint systems through systematic reviews, for example, a study of this technology in Nigeria recorded a success rate of 94% [43]. Not only did we demonstrate that fingerprint technology performed well, but we also demonstrated that it was more efficient than the traditional record-searching mechanisms at retrieving patient records from the EMRS. Data from this study add to the body of evidence of the potential of fingerprint technology to improve patient identification and matching in LMICs, which often lack unique identifiers and which experience other challenges to patient identification.

Despite performing better than traditional patient-matching approaches, the technology still failed to enroll a small number of individuals (FER 2.3%, 7/300) and to match 10.7% (32/300) of the registered individuals (FRR 10.7%, 32/300). A key reason for the failure to identify 10.7% (32/300) of the registered individuals was related to issues with the thumbprint of the individual patient (worn out thumbprint), signifying that performance of these systems could be improved by considering alternative or multiple fingers when one did not suffice. The other reasons were related to operator and technology issues, also suggesting that improving the scanner and software could improve system performance. To reduce costs, we tested the technology using only 1 scanner type (DigitalPersona U.are.U 4500; DigitalPersona, Inc) with open source software, but we recognize that there are other scanners that could perform better. There also needs to be better training of operators and improved level of machine-generated notifications to improve performance. In the COVID-19 pandemic era, it is important to not only understand how to operate these systems beyond simply recording fingerprints, but to also learn how to ensure that scanners are cleaned well and social distancing is maintained between the operator and the patient whose fingerprint is being captured. The aforementioned findings also suggest that fingerprint technology should not necessarily be seen as a replacement for traditional deterministic and probabilistic methods; rather, it should be looked upon as an additional and complementary enhancement.

This study indicated that fingerprint technology would be acceptable to patients in these settings. A surprisingly high number of participants (271/300, 90.3%) were willing to use the technology and expressed satisfaction with its use (290/300, 96.7%). This aligns with the findings from another study on adoption of fingerprint scanning during contact investigation for tuberculosis in Kampala, Uganda, where fingerprint technology was found to be feasible and acceptable [44]. A notable finding in our study was that participants’ sociodemographic characteristics and their perceptions were generally not associated with acceptability of the fingerprint technology. Previous users of the fingerprint technology biometric system were associated with more than thrice the increased odds of accepting the fingerprint biometric solution, signifying that if implemented well, fingerprint technology is likely to achieve even more acceptance with patients.

Only 15% (45/300) of the participants felt that the technology threated their privacy. Previous studies have demonstrated similar lower concern regarding privacy in LMICs. As an example, in a study evaluating authentication options for mobile health apps in younger and older adults, only approximately 10% of the participants were concerned about the storage of their face scans, whereas only 4% were concerned about the privacy requirements [45]. Users’ concerns regarding privacy tend to be paradoxical because they sometimes vary depending on the technology and user knowledge of the subject [46]. Despite our participant responses, privacy remains a key concern with fingerprint technology. In LMICs, there is a particular need for patient education and sensitization on the privacy and security of these technologies. Furthermore, there should be strong frameworks, guidelines, and standard operating procedures that cover ownership, control, and use of, as well as access to, patients’ biometric data [47]. In fact, in settings such as those of LMICs, more protections are needed, given that patients might be vulnerable simply because they may have less understanding of the technology. As previously described, ethical guidelines to consider while implementing biometrics should include, but not be limited to, the following: (1) secondary principles and rights (right of access to information and data protection principles), (2) surveillance society issues (choice, power, empowerment, transparency and accountability, consent, and communication with the user), and (3) health and hygiene concerns regarding biometrics (physical contact between people providing the biometric data and the official enrolling the individuals) [48].

This study included several limitations that deserve a mention. The technology was tested on only 1 scanner type (DigitalPersona U.are.U 4500), and only the thumbprint was used. Although the system performed very well, there is room for improvement to incorporate the capture of more fingers and to leverage the features of other scanners. In addition, infants were not catered for in this study, given the known challenges with fingerprint capture in this population [49,50]. Beyond attitudes and perceptions of patients, it would also be important to capture the perceptions of other stakeholders regarding fingerprint technologies, including providers, managers, and health ministry leaders. The next steps include further refinement of this system and working with key stakeholders to determine its role in supporting national health information systems in Kenya and other countries.

### Conclusions

Fingerprint biometric technology offers an improved patient identification mechanism compared with traditional deterministic and probabilistic techniques. Accuracy can be greatly improved when more than one finger is captured during enrollment because this captures more minutiae. The technology is also acceptable to patients in resource-limited settings. However, large-scale deployment should take ethical, legal, and social implications as well as security issues into consideration.

## Appendix

Multimedia Appendix 1 Fingerprint capture workflow.

Multimedia Appendix 2 Questionnaire to evaluate patient perception of fingerprinting patient identification system.

## Abbreviations

AMPATH: Academic Model Providing Access to Healthcare
EMRS: electronic medical record system
FAR: false acceptance rate
FER: failure to enroll rate
FRR: false rejection rate
LMIC: low- and middle-income country
