# Supplementary figures and images for: Implementation of Fingerprint Technology for Unique Patient Matching and Identification at an HIV Care and Treatment Facility in Western Kenya: Cross-sectional Study
Source: J Med Internet Res. 2021 Dec 22;23(12):e28958. doi: 10.2196/28958 (PMC8734934; doi:10.2196/28958)

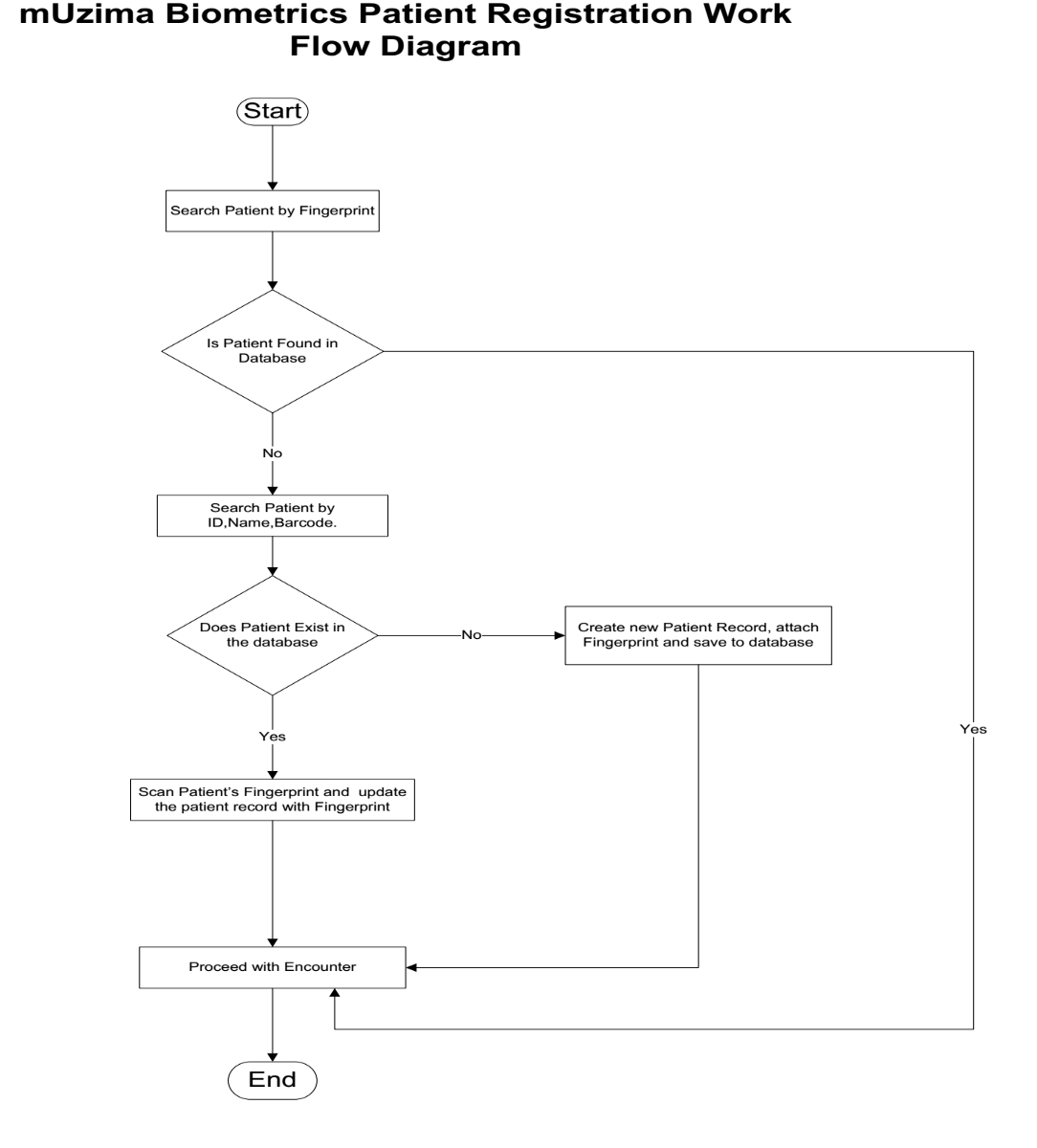

Supplement: Multimedia Appendix 1 [file jmir_v23i12e28958_app1.png]
