# Supplementary material for: Implementation of Fingerprint Technology for Unique Patient Matching and Identification at an HIV Care and Treatment Facility in Western Kenya: Cross-sectional Study
Source: J Med Internet Res. 2021 Dec 22;23(12):e28958. doi: 10.2196/28958 (PMC8734934; doi:10.2196/28958)
Supplement: Multimedia Appendix 2 [file jmir_v23i12e28958_app2.docx]

## Multimedia Appendix 2: Questionnaire to evaluate patient perceptions towards the fingerprint identification system.

| **Part A. Socio – demographic characteristics** | |
| --- | --- |
| Date of birth/Age |  |
| Gender | Male Female |
| Level of education | Primary school  High school graduate  College graduate  University graduate  Informal  Other (Specify) ………………………………………  …………………………………………………………. |
|  |  |
| Occupation/Profession | Student  Worker  Retired  Other |

| **Part B. General perception of biometric systems** | |
| --- | --- |
| Have you ever heard before about biometric authentication? | Yes No |
| Have you ever used any of these biometric systems in any hospital for authentication? | Eye/Iris Yes No  Face Yes No  Fingerprint Yes No  Voice Yes No  Other (specify) …………………………………………  …………………………………………………………. |
| In your opinion, are the current patient identifiers (IDs, Patient Numbers...) appropriate solutions for patient identification | Strongly disagree  Disagree  Agree  Strongly agree  I don’t know |
| In your opinion, do biometric solutions offer an improved solution for patient identification | Strongly disagree  Disagree  Agree  Strongly agree  I don’t know |
| **Part C. Perception of the piloted fingerprint biometric systems** | |
| How comfortable were you during registration using the fingerprint system? | Very Comfortable  Comfortable  A little Comfortable  Not Comfortable  Would rather not say |
| Are you willing to use the fingerprint biometric system in the future | Strongly disagree  Disagree  Agree  Strongly agree  I don’t know |
| What is your general perception of the system | Not at all satisfied  Not satisfied  Comment ………………………………………………  …………………………………………………………  ………………………………………………………… |
| Does the technology threaten your privacy?  Medical privacy or health privacy is the practice of keeping information about a patient confidential. | Yes No  Comment……………………………………………….  ………………………………………………………….  ………………………………………………………….. |
